# 1-(1-Hy­droxy­eth­yl)-7,8-dihydro­indolo[2,3-*a*]pyridine­[3,4-*g*]quinolizin-5(13*H*)-one (angustoline) monohydrate from *Nauclea subdita* (Rubiaceae)

**DOI:** 10.1107/S1600536811022768

**Published:** 2011-06-18

**Authors:** Sook Yee Liew, Mat Ropi Mukhtar, Khalijah Awang, Mohd Rais Mustafa, Seik Weng Ng

**Affiliations:** a Department of Chemistry, University of Malaya, 50603 Kuala Lumpur, Malaysia; b Department of Pharmacology, Faculty of Medicine, University of Malaya, 50603 Kuala Lumpur, Malaysia

## Abstract

The title compound (trivial name: angustoline monohydrate), C_20_H_17_N_3_O_2_·H_2_O, features a fused-ring system formed by one five- and four six-membered rings. The nearly planar benzimidazole portion (r.m.s. deviation = 0.008 Å) and the nearly planar 2,7-naphthyridin-1-one portion (r.m.s. deviation = 0.022 Å) of the fused-ring system are slightly twisted, with a dihedral angle of 9.47 (8)°, owing to the tetra­hedral nature of the two methyl­ene linkages in the central six-membered ring. The secondary N atom acts as a hydrogen-bond donor to the water molecule of crystallization. In the crystal, the amino and hy­droxy groups, and the water mol­ecule are engaged in hydrogen bonding, generating a three-dimensional network.

## Related literature

For the isolation of the title compound from other plants, see: Abreu & Pereira (1998 ▶, 2001 ▶); Au *et al.* (1973 ▶); Carte *et al.* (1990 ▶); Erdelmeier *et al.* (1992 ▶); Fan *et al.* (2010 ▶); Hotellier *et al.* (1975 ▶); Kakuguchi *et al.* (2009 ▶); Lin *et al.* (1988 ▶); Sun *et al.* (2008 ▶); Xuan *et al.* (2007 ▶); Zeches *et al.* (1985 ▶).
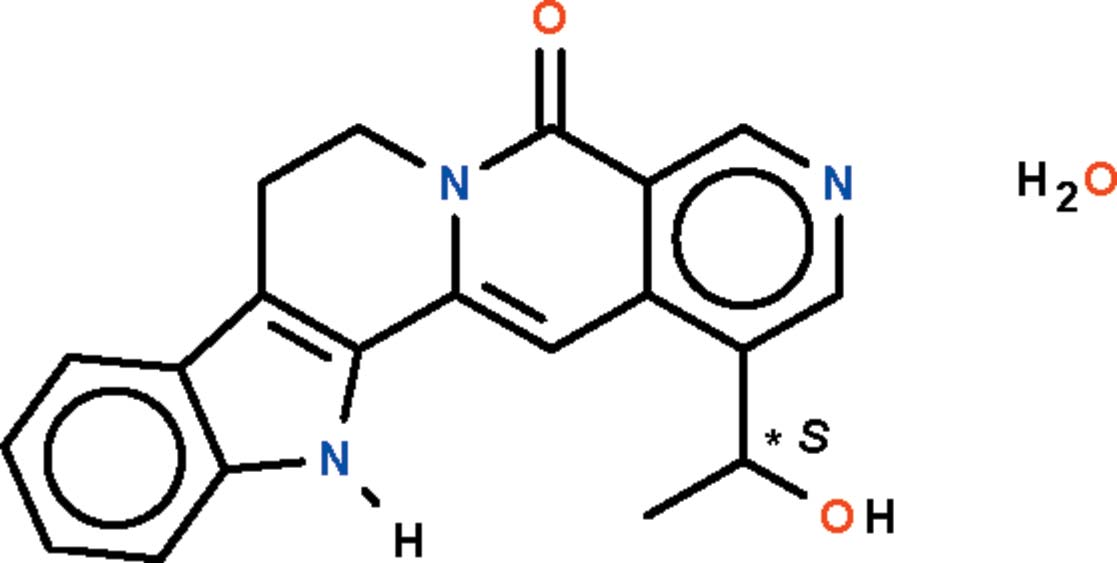


## Experimental

### 

#### Crystal data

C_20_H_17_N_3_O_2_·H_2_O
*M* *_r_* = 349.38
Monoclinic, 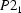

*a* = 8.8350 (3) Å
*b* = 6.7002 (2) Å
*c* = 14.7347 (4) Å
β = 103.117 (3)°
*V* = 849.48 (4) Å^3^
*Z* = 2
Cu *K*α radiation
μ = 0.76 mm^−1^
*T* = 100 K
0.30 × 0.03 × 0.03 mm

#### Data collection

Agilent SuperNova Dual with an Atlas detector diffractometer
Absorption correction: multi-scan (*CrysAlis PRO*; Agilent, 2010 ▶) *T*_min_ = 0.803, *T*_max_ = 0.978
6453 measured reflections
3252 independent reflections
3015 reflections with *I* > 2σ(*I*)
*R*_int_ = 0.027

#### Refinement

*R*[*F* ^2^ > 2σ(*F* ^2^)] = 0.036
*wR*(*F* ^2^) = 0.098
*S* = 1.02
3252 reflections
251 parameters
1 restraint
H atoms treated by a mixture of independent and constrained refinement
Δρ_max_ = 0.29 e Å^−3^
Δρ_min_ = −0.23 e Å^−3^
Absolute structure: Flack (1983 ▶), 1395 Friedel pairs
Flack parameter: 0.1 (2)

### 

Data collection: *CrysAlis PRO* (Agilent, 2010 ▶); cell refinement: *CrysAlis PRO*; data reduction: *CrysAlis PRO*; program(s) used to solve structure: *SHELXS97* (Sheldrick, 2008 ▶); program(s) used to refine structure: *SHELXL97* (Sheldrick, 2008 ▶); molecular graphics: *X-SEED* (Barbour, 2001 ▶); software used to prepare material for publication: *publCIF* (Westrip, 2010 ▶).

## Supplementary Material

Crystal structure: contains datablock(s) global, I. DOI: 10.1107/S1600536811022768/xu5242sup1.cif

Structure factors: contains datablock(s) I. DOI: 10.1107/S1600536811022768/xu5242Isup2.hkl

Additional supplementary materials:  crystallographic information; 3D view; checkCIF report

## Figures and Tables

**Table 1:** Hydrogen-bond geometry (Å, °)

*D*—H⋯*A*	*D*—H	H⋯*A*	*D*⋯*A*	*D*—H⋯*A*
O2—H2⋯N3^i^	0.97 (4)	1.77 (4)	2.732 (2)	171 (3)
O1w—H11⋯O1^ii^	0.85 (4)	2.08 (4)	2.928 (2)	169 (3)
O1w—H12⋯O2^iii^	0.83 (4)	1.95 (4)	2.762 (2)	167 (3)
N1—H1⋯O1w	0.85 (3)	2.02 (3)	2.861 (2)	177 (2)

Symmetry codes: (i) 
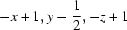
; (ii) 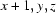; (iii) 
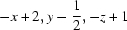
.

**Fig. 1.**
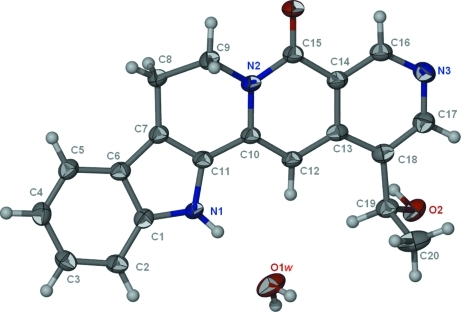
Thermal ellipsoid plot (Barbour, 2001) of C20H17N3O2.H2O at the 70% probability level; hydrogen atoms are drawn as spheres of arbitrary radius.

## supplementary crystallographic information

### Comment

The alkaloid angustoline has been isolated from a number of plants: *Camptotheca acuminata* (Carte *et al.*, 1990; Lin *et al.*, 1988), *Nauclea latifolia* (Kakuguchi *et al.*, 2009; Hotellier *et al.*, 1975), *Nauclea officinalis* (Fan *et al.*, 2010; Sun *et al.*, 2008; Xuan *et al.*, 2007), *Nauclea orientalis* (Erdelmeier *et al.*, 1992), *Nauclea pobeguinii* (Zeches *et al.*, 1985), *Sarcocephalus latifolius* (Abreu & Pereira, 1998; 2001) and *Strychnos angustiflora* (Au *et al.*, 1973).

The alkaloid is isolated in the crystalline form as a monohydrate (Scheme I). The planar benzimidazole portion and the planar 2,7-naphthyridin-1-one portion of the fused-ring system are slightly twisted [dihedral angle 9.47 (8)°] owing to the tetrahedral nature of the two methylene linkages (Fig. 1). The secondary N atom is hydrogen-bond donor to the water molecule. The amino and hydroxy groups, and the lattice water molecule are engaged in hydrogen bonding to furnish a three-dimensional network (Table 1).

### Experimental

*Nauclea subdita* (Rubiaceae) was collected from Bukit Kinta forest reserve, Chemor, Perak, Malaysia, and specimens were deposited at the Herbarium, Department of Chemistry, University of Malaya.

Dried and ground bark of *Nauclea subdita* (1.7 kg) was extracted with hexane (17 *L*) for 3 days. The hexane extract was concentrated under reduced pressure. The dried plant material was soaked in ammonium hydroxide for 2 h. It was further extracted with dichloromethane (17 *L*) for 3 days. The dichloromethane extract was concentrated under reduced pressure to give a crude alkaloid (7.1 g). A portion (6.0 g) was subjected to column chromatography on silica gel 60 GF_254_ by using a step gradient of dichloromethane and methanol. One of the fractions when further purified by using dichloromethane/methanol (95:5) afforded the pure compound, whose formulation was established by NMR spectroscopic analysis.

### Refinement

Carbon-bound H-atoms were placed in calculated positions [C—H 0.95 to 0.99 Å, *U*_iso_(H) 1.2 to 1.5*U*_eq_(C)] and were included in the refinement in the riding model approximation.

The nitrogen and oxygen bound H-atoms were located in a difference Fourier map, and were freely refined.

The Flack parameter was determined from 1395 Friedel pairs.

### Crystal data

**Table:**

C_20_H_17_N_3_O_2_·H_2_O	*F*(000) = 368
*M**_r_* = 349.38	*D*_x_ = 1.366 Mg m^−^^3^
Monoclinic, *P*2_1_	Cu *K*α radiation, λ = 1.54184 Å
Hall symbol: P 2yb	Cell parameters from 3352 reflections
*a* = 8.8350 (3) Å	θ = 3.1–74.0°
*b* = 6.7002 (2) Å	µ = 0.76 mm^−^^1^
*c* = 14.7347 (4) Å	*T* = 100 K
β = 103.117 (3)°	Prism, yellow
*V* = 849.48 (4) Å^3^	0.30 × 0.03 × 0.03 mm
*Z* = 2	

### Data collection

**Table:**

Agilent SuperNova Dual with an Atlas detector diffractometer	3252 independent reflections
Radiation source: SuperNova (Cu) X-ray Source	3015 reflections with *I* > 2σ(*I*)
Mirror	*R*_int_ = 0.027
Detector resolution: 10.4041 pixels mm^-1^	θ_max_ = 74.2°, θ_min_ = 3.1°
ω scans	*h* = −11→9
Absorption correction: multi-scan (*CrysAlis PRO*; Agilent, 2010)	*k* = −7→8
*T*_min_ = 0.803, *T*_max_ = 0.978	*l* = −17→18
6453 measured reflections	

### Refinement

**Table:**

Refinement on *F*^2^	Secondary atom site location: difference Fourier map
Least-squares matrix: full	Hydrogen site location: inferred from neighbouring sites
*R*[*F*^2^ > 2σ(*F*^2^)] = 0.036	H atoms treated by a mixture of independent and constrained refinement
*wR*(*F*^2^) = 0.098	*w* = 1/[σ^2^(*F*_o_^2^) + (0.0647*P*)^2^ + 0.0395*P*] where *P* = (*F*_o_^2^ + 2*F*_c_^2^)/3
*S* = 1.02	(Δ/σ)_max_ = 0.001
3252 reflections	Δρ_max_ = 0.29 e Å^−^^3^
251 parameters	Δρ_min_ = −0.23 e Å^−^^3^
1 restraint	Absolute structure: Flack (1983), 1395 Friedel pairs
Primary atom site location: structure-invariant direct methods	Flack parameter: 0.1 (2)

### Fractional atomic coordinates and isotropic or equivalent isotropic displacement parameters (Å^2^)

**Table:**

	*x*	*y*	*z*	*U*_iso_*/*U*_eq_	
O1	0.33730 (17)	0.5046 (2)	0.78985 (11)	0.0309 (3)	
O2	0.76411 (17)	0.6678 (2)	0.44802 (9)	0.0280 (3)	
H2	0.677 (4)	0.578 (5)	0.429 (2)	0.061 (9)*	
O1W	1.0970 (2)	0.2216 (3)	0.70030 (13)	0.0384 (4)	
H11	1.157 (4)	0.316 (6)	0.725 (2)	0.060 (10)*	
H12	1.125 (4)	0.196 (6)	0.652 (2)	0.063 (10)*	
N1	0.89459 (18)	0.0009 (2)	0.78964 (11)	0.0210 (3)	
H1	0.953 (3)	0.071 (4)	0.7637 (15)	0.022 (6)*	
N2	0.54310 (19)	0.2982 (2)	0.79266 (12)	0.0234 (3)	
N3	0.4603 (2)	0.8811 (3)	0.59777 (12)	0.0280 (4)	
C1	0.9248 (2)	−0.1772 (3)	0.83570 (12)	0.0212 (4)	
C2	1.0553 (2)	−0.3025 (3)	0.84619 (13)	0.0238 (4)	
H2A	1.1403	−0.2694	0.8195	0.029*	
C3	1.0547 (2)	−0.4757 (3)	0.89683 (12)	0.0245 (4)	
H3	1.1405	−0.5644	0.9040	0.029*	
C4	0.9304 (2)	−0.5250 (3)	0.93830 (12)	0.0256 (4)	
H4	0.9340	−0.6452	0.9729	0.031*	
C5	0.8035 (2)	−0.3999 (3)	0.92892 (12)	0.0236 (4)	
H5	0.7204	−0.4328	0.9574	0.028*	
C6	0.7989 (2)	−0.2238 (3)	0.87687 (12)	0.0214 (4)	
C7	0.6920 (2)	−0.0637 (3)	0.85452 (13)	0.0218 (4)	
C8	0.5381 (2)	−0.0264 (3)	0.87749 (14)	0.0250 (4)	
H8A	0.5377	−0.0813	0.9398	0.030*	
H8B	0.4545	−0.0924	0.8311	0.030*	
C9	0.5108 (2)	0.1974 (3)	0.87643 (14)	0.0280 (4)	
H9A	0.4015	0.2229	0.8789	0.034*	
H9B	0.5781	0.2566	0.9330	0.034*	
C10	0.6769 (2)	0.2496 (3)	0.76131 (13)	0.0209 (4)	
C11	0.7537 (2)	0.0699 (3)	0.80190 (12)	0.0214 (4)	
C12	0.7238 (2)	0.3627 (3)	0.69551 (13)	0.0205 (4)	
H12A	0.8131	0.3245	0.6740	0.025*	
C13	0.6401 (2)	0.5366 (3)	0.65911 (12)	0.0207 (4)	
C14	0.5043 (2)	0.5833 (3)	0.69001 (13)	0.0224 (4)	
C15	0.4529 (2)	0.4620 (3)	0.75959 (13)	0.0238 (4)	
C16	0.4187 (2)	0.7539 (3)	0.65684 (13)	0.0250 (4)	
H16	0.3263	0.7802	0.6775	0.030*	
C17	0.5887 (2)	0.8336 (3)	0.56645 (13)	0.0274 (4)	
H17	0.6179	0.9220	0.5230	0.033*	
C18	0.6799 (2)	0.6681 (3)	0.59251 (12)	0.0230 (4)	
C19	0.8136 (2)	0.6270 (3)	0.54537 (13)	0.0260 (4)	
H19	0.8421	0.4826	0.5537	0.031*	
C20	0.9565 (3)	0.7508 (4)	0.58426 (16)	0.0396 (5)	
H20A	1.0380	0.7178	0.5514	0.059*	
H20B	0.9938	0.7220	0.6508	0.059*	
H20C	0.9306	0.8928	0.5758	0.059*	

### Atomic displacement parameters (Å^2^)

**Table:**

	*U*^11^	*U*^22^	*U*^33^	*U*^12^	*U*^13^	*U*^23^
O1	0.0278 (7)	0.0272 (7)	0.0445 (8)	0.0050 (6)	0.0228 (6)	0.0025 (6)
O2	0.0305 (7)	0.0321 (7)	0.0238 (7)	−0.0063 (6)	0.0114 (6)	0.0003 (6)
O1W	0.0409 (9)	0.0419 (10)	0.0405 (9)	−0.0122 (7)	0.0261 (7)	−0.0102 (7)
N1	0.0210 (7)	0.0209 (8)	0.0245 (7)	0.0005 (6)	0.0125 (6)	0.0017 (6)
N2	0.0229 (8)	0.0223 (8)	0.0300 (8)	0.0018 (6)	0.0161 (6)	0.0006 (6)
N3	0.0267 (9)	0.0310 (10)	0.0272 (8)	0.0079 (7)	0.0081 (7)	0.0055 (7)
C1	0.0232 (9)	0.0216 (9)	0.0197 (8)	−0.0019 (7)	0.0068 (7)	−0.0023 (7)
C2	0.0233 (9)	0.0278 (10)	0.0222 (8)	0.0038 (7)	0.0089 (7)	−0.0024 (7)
C3	0.0263 (9)	0.0255 (10)	0.0218 (8)	0.0061 (7)	0.0057 (7)	−0.0009 (7)
C4	0.0324 (10)	0.0217 (10)	0.0234 (9)	0.0005 (8)	0.0080 (8)	0.0014 (7)
C5	0.0268 (9)	0.0231 (9)	0.0233 (8)	−0.0033 (8)	0.0107 (7)	−0.0011 (7)
C6	0.0215 (8)	0.0225 (9)	0.0218 (8)	−0.0013 (7)	0.0081 (7)	−0.0036 (7)
C7	0.0232 (9)	0.0207 (9)	0.0243 (8)	−0.0015 (7)	0.0109 (7)	−0.0025 (7)
C8	0.0239 (9)	0.0218 (10)	0.0337 (10)	0.0013 (7)	0.0161 (7)	0.0023 (8)
C9	0.0323 (10)	0.0241 (10)	0.0344 (10)	0.0022 (8)	0.0222 (9)	0.0029 (8)
C10	0.0206 (8)	0.0194 (9)	0.0255 (9)	0.0002 (7)	0.0114 (7)	−0.0032 (7)
C11	0.0218 (8)	0.0205 (9)	0.0248 (8)	0.0004 (7)	0.0112 (7)	−0.0049 (7)
C12	0.0189 (8)	0.0222 (10)	0.0232 (8)	0.0002 (7)	0.0105 (7)	−0.0024 (7)
C13	0.0188 (8)	0.0248 (10)	0.0198 (8)	−0.0010 (7)	0.0070 (7)	−0.0036 (6)
C14	0.0204 (8)	0.0242 (9)	0.0240 (8)	−0.0003 (7)	0.0082 (7)	−0.0011 (7)
C15	0.0224 (9)	0.0214 (10)	0.0305 (9)	0.0003 (7)	0.0122 (7)	−0.0026 (7)
C16	0.0218 (9)	0.0287 (10)	0.0266 (9)	0.0021 (7)	0.0101 (7)	−0.0002 (8)
C17	0.0273 (10)	0.0328 (11)	0.0233 (9)	0.0028 (8)	0.0086 (8)	0.0062 (7)
C18	0.0223 (9)	0.0277 (10)	0.0201 (8)	−0.0006 (7)	0.0073 (7)	−0.0015 (8)
C19	0.0251 (9)	0.0320 (11)	0.0235 (8)	0.0017 (7)	0.0107 (7)	0.0020 (7)
C20	0.0277 (10)	0.0625 (15)	0.0319 (11)	−0.0070 (10)	0.0134 (8)	−0.0115 (10)

### Geometric parameters (Å, °)

**Table:**

O1—C15	1.237 (2)	C7—C11	1.375 (3)
O2—C19	1.428 (2)	C7—C8	1.495 (2)
O2—H2	0.97 (4)	C8—C9	1.518 (3)
O1W—H11	0.85 (4)	C8—H8A	0.9900
O1W—H12	0.83 (4)	C8—H8B	0.9900
N1—C1	1.369 (2)	C9—H9A	0.9900
N1—C11	1.378 (2)	C9—H9B	0.9900
N1—H1	0.85 (3)	C10—C12	1.366 (2)
N2—C15	1.379 (3)	C10—C11	1.443 (3)
N2—C10	1.402 (2)	C12—C13	1.419 (3)
N2—C9	1.490 (2)	C12—H12A	0.9500
N3—C16	1.328 (3)	C13—C14	1.412 (2)
N3—C17	1.356 (3)	C13—C18	1.421 (3)
C1—C2	1.406 (3)	C14—C16	1.396 (3)
C1—C6	1.418 (2)	C14—C15	1.459 (3)
C2—C3	1.380 (3)	C16—H16	0.9500
C2—H2A	0.9500	C17—C18	1.373 (3)
C3—C4	1.412 (3)	C17—H17	0.9500
C3—H3	0.9500	C18—C19	1.525 (2)
C4—C5	1.381 (3)	C19—C20	1.511 (3)
C4—H4	0.9500	C19—H19	1.0000
C5—C6	1.403 (3)	C20—H20A	0.9800
C5—H5	0.9500	C20—H20B	0.9800
C6—C7	1.418 (3)	C20—H20C	0.9800
			
C19—O2—H2	102.3 (19)	H9A—C9—H9B	107.7
H11—O1W—H12	104 (3)	C12—C10—N2	121.26 (17)
C1—N1—C11	107.94 (15)	C12—C10—C11	124.55 (16)
C1—N1—H1	129.4 (16)	N2—C10—C11	114.15 (15)
C11—N1—H1	122.2 (16)	C7—C11—N1	109.94 (17)
C15—N2—C10	122.12 (15)	C7—C11—C10	124.60 (16)
C15—N2—C9	116.72 (15)	N1—C11—C10	125.37 (16)
C10—N2—C9	120.11 (16)	C10—C12—C13	120.54 (16)
C16—N3—C17	116.71 (17)	C10—C12—H12A	119.7
N1—C1—C2	129.66 (17)	C13—C12—H12A	119.7
N1—C1—C6	108.67 (16)	C14—C13—C12	117.92 (16)
C2—C1—C6	121.66 (17)	C14—C13—C18	116.52 (17)
C3—C2—C1	117.27 (17)	C12—C13—C18	125.56 (16)
C3—C2—H2A	121.4	C16—C14—C13	120.08 (16)
C1—C2—H2A	121.4	C16—C14—C15	118.13 (16)
C2—C3—C4	121.95 (17)	C13—C14—C15	121.74 (17)
C2—C3—H3	119.0	O1—C15—N2	120.96 (17)
C4—C3—H3	119.0	O1—C15—C14	122.63 (18)
C5—C4—C3	120.55 (18)	N2—C15—C14	116.38 (16)
C5—C4—H4	119.7	N3—C16—C14	123.12 (16)
C3—C4—H4	119.7	N3—C16—H16	118.4
C4—C5—C6	119.14 (17)	C14—C16—H16	118.4
C4—C5—H5	120.4	N3—C17—C18	125.27 (19)
C6—C5—H5	120.4	N3—C17—H17	117.4
C5—C6—C1	119.41 (17)	C18—C17—H17	117.4
C5—C6—C7	134.32 (17)	C17—C18—C13	118.21 (17)
C1—C6—C7	106.26 (16)	C17—C18—C19	118.98 (17)
C11—C7—C6	107.19 (16)	C13—C18—C19	122.73 (17)
C11—C7—C8	121.06 (17)	O2—C19—C20	108.34 (17)
C6—C7—C8	131.75 (17)	O2—C19—C18	109.32 (16)
C7—C8—C9	108.24 (15)	C20—C19—C18	113.28 (16)
C7—C8—H8A	110.1	O2—C19—H19	108.6
C9—C8—H8A	110.1	C20—C19—H19	108.6
C7—C8—H8B	110.1	C18—C19—H19	108.6
C9—C8—H8B	110.1	C19—C20—H20A	109.5
H8A—C8—H8B	108.4	C19—C20—H20B	109.5
N2—C9—C8	113.37 (16)	H20A—C20—H20B	109.5
N2—C9—H9A	108.9	C19—C20—H20C	109.5
C8—C9—H9A	108.9	H20A—C20—H20C	109.5
N2—C9—H9B	108.9	H20B—C20—H20C	109.5
C8—C9—H9B	108.9		
			
C11—N1—C1—C2	−177.98 (19)	N2—C10—C11—C7	10.4 (3)
C11—N1—C1—C6	0.94 (19)	C12—C10—C11—N1	8.9 (3)
N1—C1—C2—C3	−179.93 (17)	N2—C10—C11—N1	−173.35 (17)
C6—C1—C2—C3	1.3 (3)	N2—C10—C12—C13	1.9 (3)
C1—C2—C3—C4	−1.2 (3)	C11—C10—C12—C13	179.54 (17)
C2—C3—C4—C5	0.3 (3)	C10—C12—C13—C14	−2.7 (3)
C3—C4—C5—C6	0.6 (3)	C10—C12—C13—C18	178.32 (18)
C4—C5—C6—C1	−0.5 (3)	C12—C13—C14—C16	179.44 (17)
C4—C5—C6—C7	−178.7 (2)	C18—C13—C14—C16	−1.5 (3)
N1—C1—C6—C5	−179.48 (16)	C12—C13—C14—C15	2.1 (3)
C2—C1—C6—C5	−0.5 (3)	C18—C13—C14—C15	−178.81 (17)
N1—C1—C6—C7	−0.8 (2)	C10—N2—C15—O1	−178.32 (18)
C2—C1—C6—C7	178.23 (17)	C9—N2—C15—O1	−10.0 (3)
C5—C6—C7—C11	178.7 (2)	C10—N2—C15—C14	−0.1 (3)
C1—C6—C7—C11	0.3 (2)	C9—N2—C15—C14	168.16 (17)
C5—C6—C7—C8	−1.8 (4)	C16—C14—C15—O1	0.0 (3)
C1—C6—C7—C8	179.82 (19)	C13—C14—C15—O1	177.41 (18)
C11—C7—C8—C9	−26.5 (3)	C16—C14—C15—N2	−178.11 (18)
C6—C7—C8—C9	154.1 (2)	C13—C14—C15—N2	−0.7 (3)
C15—N2—C9—C8	147.19 (18)	C17—N3—C16—C14	3.0 (3)
C10—N2—C9—C8	−44.3 (3)	C13—C14—C16—N3	−1.6 (3)
C7—C8—C9—N2	47.4 (2)	C15—C14—C16—N3	175.87 (18)
C15—N2—C10—C12	−0.5 (3)	C16—N3—C17—C18	−1.5 (3)
C9—N2—C10—C12	−168.38 (18)	N3—C17—C18—C13	−1.4 (3)
C15—N2—C10—C11	−178.32 (17)	N3—C17—C18—C19	175.34 (19)
C9—N2—C10—C11	13.8 (2)	C14—C13—C18—C17	2.8 (3)
C6—C7—C11—N1	0.2 (2)	C12—C13—C18—C17	−178.17 (18)
C8—C7—C11—N1	−179.32 (17)	C14—C13—C18—C19	−173.81 (17)
C6—C7—C11—C10	176.94 (17)	C12—C13—C18—C19	5.2 (3)
C8—C7—C11—C10	−2.6 (3)	C17—C18—C19—O2	−41.1 (2)
C1—N1—C11—C7	−0.7 (2)	C13—C18—C19—O2	135.53 (18)
C1—N1—C11—C10	−177.41 (17)	C17—C18—C19—C20	79.8 (2)
C12—C10—C11—C7	−167.33 (19)	C13—C18—C19—C20	−103.6 (2)

### Hydrogen-bond geometry (Å, °)

**Table:**

*D*—H···*A*	*D*—H	H···*A*	*D*···*A*	*D*—H···*A*
O2—H2···N3^i^	0.97 (4)	1.77 (4)	2.732 (2)	171 (3)
O1w—H11···O1^ii^	0.85 (4)	2.08 (4)	2.928 (2)	169 (3)
O1w—H12···O2^iii^	0.83 (4)	1.95 (4)	2.762 (2)	167 (3)
N1—H1···O1w	0.85 (3)	2.02 (3)	2.861 (2)	177 (2)

Symmetry codes: (i) −*x*+1, *y*−1/2, −*z*+1; (ii) *x*+1, *y*, *z*; (iii) −*x*+2, *y*−1/2, −*z*+1.
